# Lack of evidence for the effectiveness or safety of over-the-counter cannabidiol products

**DOI:** 10.1177/2045125320954992

**Published:** 2020-09-09

**Authors:** Edward Chesney, Philip McGuire, Tom P. Freeman, John Strang, Amir Englund

**Affiliations:** Department of Psychosis Studies, Institute of Psychiatry, Psychology and Neuroscience, King’s College London, De Crespigny Park, London SE5 8AF, UK; South London and Maudsley NHS Foundation Trust, London, UK; Department of Psychosis Studies, Institute of Psychiatry, Psychology & Neuroscience, King’s College London, London, UK; National Institute for Health Research, Maudsley Biomedical Research Centre, London, UK; Addiction and Mental Health Group (AIM), Department of Psychology, University of Bath, London, UK; Addictions Department, Institute of Psychiatry, Psychology and Neuroscience, King’s College London, London, UK; South London and Maudsley NHS Foundation Trust, London, UK; Addictions Department, Institute of Psychiatry, Psychology and Neuroscience, King’s College London, London, UK; Addictions Department, Institute of Psychiatry, Psychology and Neuroscience, King’s College London, London, UK

**Keywords:** cannabidiol, CBD, cannabis oil, over the counter, health supplement, safety, efficacy

## Abstract

Over the past 5 years, public interest in the potential health benefits of cannabidiol (CBD) has increased exponentially, and a wide range of over-the-counter (OTC) preparations of CBD are now available. A substantial proportion of the population appears to have used these products, yet the extent to which they are effective or safe is unclear. We reviewed the evidence for whether CBD has significant pharmacological and symptomatic effects at the doses typically found in OTC preparations. We found that most of the evidence for beneficial effects is derived from studies of pure, pharmaceutical grade CBD at relatively high doses. Relatively few studies have examined the effect of OTC CBD preparations, or of CBD at low doses. Thus, at present, there is little evidence that OTC CBD products have health benefits, and their safety has not been investigated. Controlled trials of OTC and low-dose CBD preparations are needed to resolve these issues.

## Introduction

Cannabidiol (CBD) can be bought as an over-the-counter (OTC) food supplement in a variety of forms, such as capsules, oils, cigarettes or an e-liquid.^1,2^ It is also sold in drinks, foods and cosmetics, such as hand creams.^3^ However, despite the widespread availability of these products, the safety and efficacy of OTC CBD are unclear.^1,4^ We sought to address this issue in the present review.

**Figure 1.**
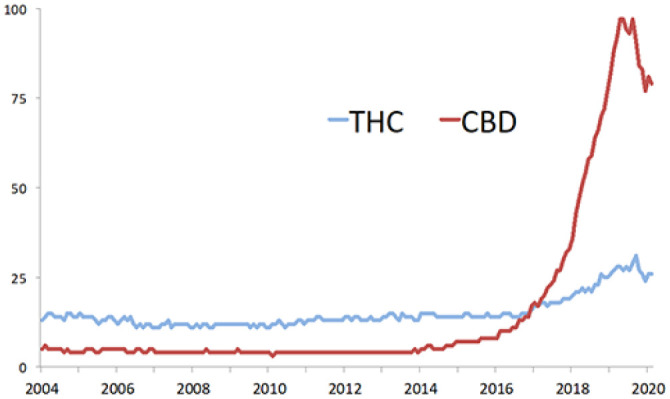
Google search trends for the terms ‘THC’ [tetrahydrocannabinol] and ‘CBD’ [cannabidiol], worldwide January 2004–February 2020.

CBD was isolated in the late 1930s^5,6^ and its chemical structure was first described by Mechoulam in 1963.^7^ Although it can be produced synthetically, the CBD in OTC products is almost always derived from plant extracts.^8^ Studies of pure, pharmaceutical grade CBD in healthy volunteers indicate that it is not intoxicating, but may have limited subjective effects at very high doses.^9,10^ Neuroimaging studies have shown that it has effects on brain activity,^11,12^ and can modulate both the endocannabinoid and other neurotransmitter function in both volunteers and patients.^13–15^ Moreover, data from experimental medicine studies and clinical trials suggest that pharmaceutical grade CBD can reduce anxiety^16,17^ and psychotic symptoms,^18^ and has beneficial effects in addiction^19^ and childhood epilepsy.^20^ Endocannabinoid signalling also has a role in regulating the immune system, and CBD also has potential as an anti-inflammatory agent.^21^ This research has led to an exponential increase in consumer interest in CBD over the past 5 years (Figure 1). It is estimated that OTC CBD health supplements have been used by one in 10 adults in the United Kingdom (UK).^22^ Retail sales data indicate that in 2019, OTC CBD was bought by 1.3 million users who spent an estimated £300 million, more than the vitamin D and vitamin C markets combined.^22^ However, almost all of the data that have driven this interest have been derived from studies of pharmaceutical grade, rather than OTC CBD. In this paper, we explore the pharmacokinetics, metabolism, mechanism of action and therapeutic and adverse effects of CBD, as well as addressing the relevant safety, labelling and legal considerations for OTC CBD products.

## The administration, dose and pharmacokinetics of OTC CBD

**Figure 2.**
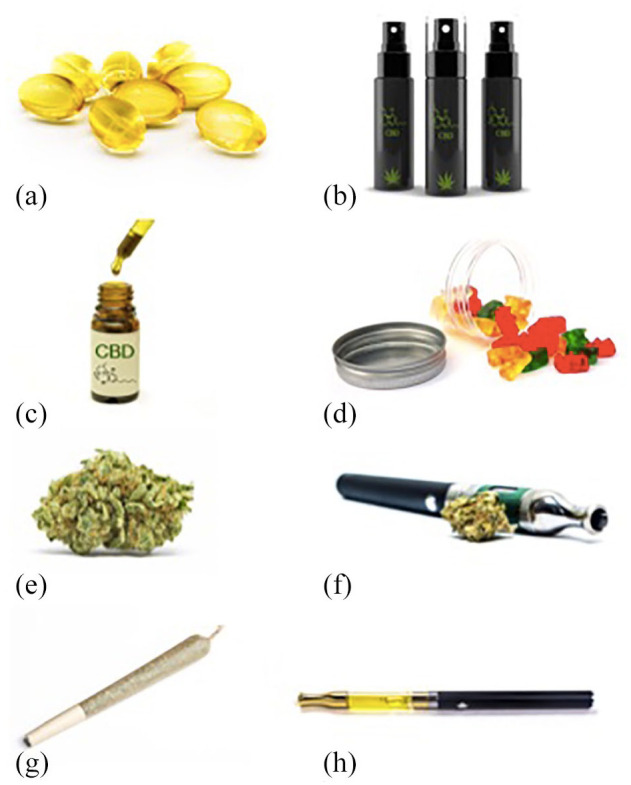
OTC CBD products: (a) Capsules, (b) Spray, (c) Oil and dropper, (d) Gummies, a type of ‘edible’. (e) Dried plant material, (f) A dry vaporizer, (g) Joint, and (h) e-Liquid vaporizer

CBD can be administered *via* the oral, oromucosal or inhaled routes.^23^ Novel methods such as transdermal administration (using a permeation enhancer to increase absorption) have also been tested.^24^ Most OTC preparations are sold as capsules or oils, which are administered using a dropper or spray (Figure 2). Cannabis plant material (of which some strains, such as hemp, contain high concentrations of CBD and minimal Δ^9^-tetrahydrocannabinol [Δ^9^-THC]) can be inhaled, either by burning or using a vaporiser to heat dry plant material. Some vaporiser models can also use e-liquids containing CBD.^2^

In clinical trials, a typical CBD dose might be approximately 1000 mg/day, often taken in two divided doses.^4^ For childhood epilepsy syndromes the recommended dose is 10–20 mg/kg/day. However, most OTC preparations contain much smaller amounts of CBD. For example, a popular UK health food shop sells CBD capsules and edibles from 5 to 20 mg per dose, and sprays and oils from 2 to 8 mg per dose.^25^ The maximum recommended dose for these products is normally below 30 mg/day. In some countries there is more variability between products; a US study found that the concentration of products bought online was between 0.10 mg/mL and 655 mg/mL (median: 9.45 mg/mL).^8^

The pharmacokinetics of CBD have been systematically reviewed by Millar *et al.*^23^ They examined 24 studies, most of which assessed the administration of CBD at doses of 5–20 mg/day, which corresponds to the doses typically found in OTC preparations. With oral administration, single doses of 5.4 and 10 mg CBD achieved peak serum concentrations (C_max_) of 0.9 and 2.5 ng/ml. The time to maximum concentration (T_max_) was approximately 1 h, with a half-life between 1 and 3 h. CBD is highly lipophilic, and its oral bioavailability is highly dependent on food as a high-fat meal can quadruple plasma exposure.^26^

For oromucosal administration, 10–20 mg CBD had a C_max_ of approximately 2–4 ng/mL, a T_max_ between 1 and 4 h, and a half-life between 1.4 and 11 h.^23^ In a study by Stott *et al.*,^27^ single doses of 5, 10 and 20 mg CBD achieved C_max_ of 0.4, 1.2 and 2.2 ng/mL. After 9 days of regular dosing, the corresponding C_max_ were 0.5, 1.1 and 3.2 ng/mL. These results indicate that at these doses, the C_max_ of CBD is dose-dependent and that CBD does not accumulate with regular dosing. As with oral administration, the bioavailability of oromucosal CBD is 3–5 times higher with food,^28^ suggesting that even with oromucosal administration, a significant proportion of absorption is gastrointestinal.

The only study of inhaled CBD identified by the review of Millar *et al.* found that 19 mg CBD achieved a much higher C_max_ (110 ng/ml) immediately after smoking, before falling to 10.2 ng/ml an hour later.^29^ Unlike oral (and oromucosal) administration, inhalation avoids first-pass metabolism, an important feature as CBD undergoes extensive metabolism in the liver before reaching central targets.

## Metabolism

CBD is metabolized by hepatic cytochrome P450 enzymes to form 7-hydroxy-CBD, 7-carboxy-CBD and 6-hydroxy-CBD as well as other minor metabolites.^29^ The two enzymes with the most substantial role are CYP2C19 and CYP3A4.^30,31^ While there has been some research in animal models, to date there have been no studies examining the pharmacological actions of CBD metabolites in humans (for a review, see Ujváry and Hanuš).^32^ However, there is evidence from human studies that the metabolism of CBD can lead to clinically relevant drug interactions, in particular *via* inhibition of CYP2C19. The antiepileptic medication clobazam is often prescribed as a treatment for childhood epilepsy syndromes. CBD can increase levels of N-desmethylclobazam, which itself is a potent antiepileptic,^33^ by approximately 5-fold.^34^ This interaction with clobazam may account for the increased risk of sedation and pneumonia seen in clinical trials of CBD in Dravet and Lennox–Gastaut syndrome.^4^ It is important to note that the doses used in clinical studies (at least 5 mg/kg) are much higher than in OTC preparations (5–20 mg/day). However, there are (non-peer-reviewed) reports that drug interactions may occur at doses as low as 1 mg/kg CBD.^35^ CYP2C19 also has a role in the metabolism of tricyclic antidepressants, selective serotonin reuptake inhibitors, proton pump inhibitors, clopidogrel, gliclazide, propranolol, and other antiepileptic medications such as diazepam and phenytoin.^36^ CBD can also affect the activity of several other CYP enzymes such as 2D6, 2C9 and 3A4, which may lead to additional interactions with antiepileptics, antipsychotics and other drugs.^37,38^ There have been case reports of drug–drug interactions with both warfarin^39^ and methadone.^40^

## Mechanism of action

CBD has limited affinity for the orthosteric binding site of cannabinoid (CB) receptors and therefore has little direct activity. However, it may act as a negative allosteric modulator at CB1 and CB2 receptors, reducing their response to agonists such as Δ^9^-THC, anandamide and 2-arachidonoylglycerol.^41,42^ Anandamide and 2-arachidonoylglycerol are endogenous CB receptor ligands known as endocannabinoids. Anandamide is metabolised by fatty acid amide hydrolase (FAAH) which can also be inhibited by CBD.^43^ Other potential mechanisms of action include agonism of transient receptor potential (TRP) channels, partial agonism of dopamine D2 receptors and serotonin 5HT_1A_ receptors, positive allosteric modulation of μ and δ-opioid receptors, inhibition of adenosine reuptake, competitive antagonism of G protein-coupled receptor 55 (GPR55) and inhibition of inflammatory cytokines *via* peroxisome proliferator-activated receptor-gamma (PPARγ) receptors.^44–47^

Because the doses of CBD in OTC preparations are much lower than in pharmaceutical grade preparations, they are extremely unlikely to produce plasma concentrations above 10 ng/ml.^23^ However, for most of the putative molecular targets described above, CBD has a half-maximal effective or inhibitory concentration (EC_50_/IC_50_) in the micromolar range (Table 1).^47^ The only target where CBD may have activity in the low nanomolar range is the negative allosteric site of CB2 receptors (IC_50_: 2–8 nM).^48^

**Table 1.** The affinity and half-maximal concentrations for the putative molecular targets of CBD.

Target	EC_50_/IC_50_, SEM (nM)	K_i_ (nM)
CB1 orthosteric site	–	3245 nM
CB1 allosteric site		304
CB2 orthosteric site	503 ± 2080	3612 ± 1382
CB2 allosteric site	2–8	3.6 ± 0.3
FAAH	19,800 ± 4770	–
Anandamide transporter	10,200 ± 3030	
TRPM8	70 ± 14	–
TRPA1	100 ± 10	–
TRPV1	1900 ± 802	3600 ± 200
TRPV2	12,200 ± 9770	–
D2	66 ± 20	11
Adenosine uptake (ENT1)	122	–
GPR55	433 ± 43	–
PPARγ receptors	5000	–
α3 Glycine receptor	11,000	–
5HT1A	–	16,000

EC_50_, half-maximal effective concentration; IC_50_, half-maximal inhibitory concentration; K_i_, inhibitory constant, the affinity of the ligand for a receptor.

Data from McPartland *et al.*,^49^ Martinez-Pinilla *et al.*,^48^ Laprairie *et al.*^41^ and Seeman.^47^

## Allosteric modulation of CB2 receptors

The activity of CBD at CB2 receptors is highly complex. Similar to CB1 receptors, CB2 receptors are G-protein coupled receptors which regulate signalling cascades such as cAMP, β-arrestins and extracellular signal-regulated kinases.^50^ CB receptors also demonstrate functional selectivity where ‘biased’ agonists are able to activate certain downstream pathways preferentially.^51^ Navarro *et al.* examined the effect of CBD on biased activity at CB1 and CB2 receptors as well as CB1–CB2 receptor heteromers. They found that, at a concentration of 100 nM, CBD ‘profoundly affected the agonist effect’ of the endogenous ligand anandamide at CB2 receptors.^51^ While the concentration of CBD was an order of magnitude higher than what can be achieved by OTC preparations, these results indicate that meaningful activity at CB2 receptors is at least theoretically possible.

CB2 receptors may be present throughout the central nervous system. In human post-mortem studies they have been identified in cerebellar microglial cells,^52^ and animal models have also suggested that they may be present in the cortex, hippocampus, ventral tegmental area and nucleus accumbens.^53^ CB2 receptors may have a neuroprotective role and have therefore been proposed as a target for several neurological and psychiatric disorders. Animal models of Alzheimer’s disease, traumatic brain injury, addiction, anxiety and depression have all found that CB2 agonists can be beneficial.^53^ In humans, it has also been shown that a common CB2 receptor variant, Q63R, which reduces its activity, is associated with eating disorders.^54^

## CBD enantiomers: natural *versus* synthetic CBD

The cannabis plant only produces the (–) enantiomer of CBD, whereas both the (+)-CBD and (–)-CBD enantiomers can be made synthetically.^55^ Data from studies that have examined the binding affinity of CBD enantiomers and their metabolites for CB receptors are shown in Table 2. The (+) enantiomer and its metabolites all have a considerably higher affinity for CB receptors than the (–) enantiomer, including at concentrations in the low nanomolar range.^55–57^ To date, there have been no studies comparing the effects of different CBD enantiomers or their metabolites in humans, and *in vivo* research has only been completed in mice.^57^ All OTC preparations use CBD derived from a purified cannabis extract, rather than synthetic CBD. Therefore, based on the existing receptor affinity data, the pharmacological activity of OTC preparations appears to be limited to the allosteric modulation of CB2 receptors.

**Table 2.** The binding affinity of (+) and (–) enantiomers of CBD to CB receptors.

Compound	CB1 K_i_ (nM)	CB2 K_i_ (nM)
(–)-CBD	>10,000	>10,000
(–)-7-OH-CBD	>10,000	>10,000
(–)-7-COOH-CBD	>10,000	>10,000
(+)-CBD	842 ± 36	203 ± 16
(+)-7-OH-CBD	5.3 ± 0.5	101 ± 5
(+)-7-COOH-CBD	13.2 ± 0.4	322 ± 16

Data from Morales *et al.*,^55^ Bisogno *et al.*^56^ and Fride *et al.*^57^

## Excipients, cannabis oils and the entourage hypothesis

In general, OTC preparations do not contain pure CBD.^8^ Most contain additional constituents and, if used clinically, are often described as ‘cannabis-based medicine extracts’ (CBMEs). To produce CBD oil, a solvent is added to cannabis plant material, extracting cannabinoids which are then added to an edible oil.^58^ To purify the extract, a process called ‘winterization’ is used, in which it is cooled and fractionated so that unwanted compounds with different melting points are removed.^59^ However, not all impurities are removed, and the oil may also contain other cannabinoids, such as Δ^9^-THC, tetrahydrocannabinolic acid, cannabigerol and cannabinol as well as terpenoids such as limonene, myrcene, α-pinene and linalool. It has been suggested that the combination of CBD with other cannabinoid and terpenoid compounds may lead to synergistic or complementary effects, producing an ‘entourage effect’ that is greater than that of CBD alone.^60^ However, experimental studies have consistently failed to provide evidence for such an effect.^61–64^ Nevertheless, the concept of the ‘entourage effect’ remains popular and is used to promote the health benefits of OTC supplements.^65^

## Clinical effects of CBD at OTC doses

Consumer interest in CBD has partly been driven by positive results from clinical trials of pharmaceutical grade CBD. CBD has been assessed in randomized controlled clinical trials in rare childhood epilepsies,^20,66–68^ schizophrenia,^18,69^ type II diabetes,^70^ fatty liver disease,^71^ Crohn’s disease,^72^ Parkinson’s disease,^73^ Huntington’s disease,^74^ and cannabis dependence.^19^ The results from the trials in childhood epilepsy have led to its licensing as a treatment for Lennox–Gastaut syndrome and Dravet syndrome. The only other licensed medicine that contains CBD is the oromucosal spray nabiximols (Sativex), which contains similar quantities of Δ^9^-THC and CBD, and is approved as a treatment for spasticity in multiple sclerosis. Nabiximols has also been trialled as a treatment for pain, and for nausea and vomiting.^75^ However, it is unclear if the effectiveness of nabiximols is related to the presence of CBD.

Most of the clinical trials with positive findings have studied the effects of CBD at doses considerably higher than in OTC preparations (>300 mg/day or 10 mg/kg/day).^4^ Nevertheless, at least eight randomized placebo controlled clinical trials have examined the effects of CBD (or CBD predominant CBME) at low doses (Table 3). Most of these studies were small and reported limited efficacy with few adverse effects. An exception was the CAMS trial in multiple sclerosis, which examined the effects of a CBD-predominant CBME, as well as a Δ^9^-THC-based medicine on spasticity^76^ and urinary symptoms in 630 patients.^77^ Although some subjective outcomes were better with CBME, there was no evidence for an effect on objective outcomes, such as the Ashworth scale for spasticity and a walking test. Moreover, 77% of those in the CBME arm guessed that they had received active treatment, compared to only 50% in the placebo group, suggesting that blinding was inadequate. In a sub-study of lower urinary tract symptoms (CAMS-LUTS),^77^ the CBME arm achieved a 25% reduction in episodes of urge incontinence compared to placebo (*p* = 0.005), although there were no changes in urodynamic studies. The lack of blinding in these studies is a particular concern, as there can be a strong allegiance bias among patients in favour of cannabis-based medicines. This phenomenon was highlighted in another study involving children prescribed cannabinoids for epilepsy: parents of children who had moved from out of state to access the programme were twice as likely as local parents to report benefits.^78^

**Table 3.** Placebo controlled clinical studies of low dose CBD and CBD predominant cannabis-based medicine extracts.

Study	Patient group	Participants	Design	Low-dose CBD arms	Comparison arms	Type of CBD & administration	Efficacy of low-dose CBD	Adverse effects
Naftali *et al.*^72^	Crohn’s disease	19	Parallel groups 8 weeks	CBD 10 mg BD (*n* = 10)	Placebo (*n* = 9)	Natural extract Oral	None	None
Tomida *et al.*^79^	Ocular hypertension & glaucoma	6	4-arm crossover Single dose	CBD 20 mg CBD 40 mg	Placebo Δ^9^-THC 5 mg	Natural extract Sublingual	Transient elevation in intraocular pressure with CBD 40 mg	Minimal
Notcutt^80^	Chronic pain	34	‘N of 1’ design 4 arms 8 × 1 week crossovers	CBME (>95% CBD) 2.5 mg as required (median 8/day)	Δ^9^-THC 2.5 mg Δ^9^-THC 2.5 mg/CBD 2.5 mg Placebo	Natural extract Sublingual	No efficacy for pain Subjective improvement in sleep quality	Minimal
Carlini and Cunha^81^ Experiment 1	Healthy volunteers	10	Parallel groups Single dose	CBD 10 mg (*n* = 2) CBD 40 mg (*n* = 2)	Placebo (*n* = 2) CBD 80 mg (*n* = 2) CBD 160 mg (*n* = 2)	Natural extract Oral	N/A	None
Carlini and Cunha^81^ Experiment 4	Healthy volunteers	4	Mixed 20 days	CBD 5 mg BD oral (*n* = 3)	Placebo (*n* = 1)	Natural extract Oral	N/A	Somnolence in 2/3 CBD participants
Carlin and Cunha^81^ Clinical trial as a hypnotic drug	Insomniacs	15	Crossover Single dose at night	CBD 40 mg	CBD 80 mg CBD 160 mg Nitrazepam 5 mg Placebo	Natural extract Oral	Reduced dream recall at 40 mg. No changes to other sleep parameters	None
CAMS^76^	Spasticity in multiple sclerosis	630	Parallel groups 15 weeks	CBME (>95% CBD) 1.25 mg, 2–5 capsules BD (*n* = 211)	Δ^9^-THC 2.5 mg, 4–10 capsules/day (*n* = 206) Placebo, 2–5 capsules BD (*n* = 213)	Natural extract Oral	No difference in Ashworth score of spasticity or walking speed. Subjective improvement in pain and spasticity NB. Issues with blinding	Constipation, diarrhoea, increased appetite
CAMS-LUTS^77^	Urge incontinence in multiple sclerosis	522	Parallel groups 15 weeks	CBME (>95% CBD) 1.25 mg, 2–5 capsules BD (*n* = 181)	Δ^9^-THC 2.5 mg, 2–5 capsules BD (*n* = 174) Placebo, 2–5 capsules BD (*n* = 167)	Natural extract Oral	25% reduction (*p* = 0.005) in incontinence relative to placebo NB. Issues with blinding	None
Morgan *et al.*^82^	Smokers	24	Parallel groups 7 days	CBD 400 μg PRN (*n* = 12)	Placebo (*n* = 12)	Synthetic Inhaled	Reduced cigarette consumption	None

BD, twice a day.

## Can low doses of CBD moderate the effects of Δ^9^-THC on psychotic symptoms?

An important question for recreational cannabis users is whether low doses of CBD can moderate the adverse effects of Δ^9^-THC, particularly on psychotic and anxiety symptoms.^83^ Observational research has suggested that CBD may reduce psychotic symptoms and cognitive impairments in some regular cannabis users.^84^ In a small experimental study in healthy volunteers, Bhattacharya *et al.* found that pre-treatment with a 5 mg dose of intravenous (IV) CBD attenuated the severity of psychotic symptoms and anxiety subsequently induced by IV Δ^9^-THC (1.25 mg).^85^ Similarly, a study in a larger sample of volunteers that used oral (rather than IV) CBD (600 mg), also found that pretreatment with CBD attenuated the induction of psychotic symptoms by IV Δ^9^-THC (1.5 mg).^86^ Another study using inhaled CBD (16 mg) did not find an effect on psychotic symptoms,^87^ but in that case, Δ^9^-THC alone had no effect on psychotic symptoms relative to placebo, precluding the detection of a modulatory effect of CBD. Thus, evidence that CBD may reduce the adverse effects of Δ^9^-THC is limited to studies that used relatively high doses of CBD. It is not known if low doses of CBD have a similar effect.

## Effects of CBD on anxiety

In an online survey of 2400 medicinal cannabis users from the United States (US), the majority cited a range of indications for their use of cannabis products including anxiety (66%), insomnia (59%), joint pain and inflammation (49%), depression (44%), migraines (32%), muscle tension or strain (32%), or severe and chronic pain (28%).^88^ An Australian survey of 1388 medicinal cannabis users listed a similar range of indications, including pain (62%), sleep (49%), mental health (45%), gastrointestinal disorders (13%), neurological disorders (11%) and cancer (8%).^89^ However, the evidence base for most of these putative indications is limited, with the exception of anxiety. A recent systematic review identified six randomized pre-clinical studies in this area.^90^ Most used doses of 300 mg or higher, and two examined a range of different doses. The first of these asked 60 healthy volunteers to complete a simulated public speaking test after pre-treatment with either CBD (100, 300 and 900 mg), clonazepam (1 mg) or placebo.^16^ In the second, 57 healthy volunteers completed a similar task after pre-treatment with a single dose of either CBD (150, 300, 600 mg) or placebo.^17^ In both studies, only the 300 mg dose of CBD reduced anxiety symptoms. The lower doses (100 often 150 mg) had no effect suggesting that OTC preparations (10–20 mg) are unlikely to be effective.

## Adverse effects and safety

A recent meta-analysis of randomized clinical trials found that, compared to placebo, CBD is associated with reduced tolerability and increased risks of pneumonia, abnormal liver function tests, decreased appetite, diarrhoea, somnolence and sedation.^4^ However, after excluding studies in epilepsy, in which CBD has pharmacokinetic interactions with clobazam and sodium valproate, diarrhoea was the only adverse event present. In this meta-analysis, the majority of the studies used doses above 300 mg/day or 10 mg/kg/day. It is therefore not known whether CBD can cause adverse events at OTC doses.

## Content of OTC preparations

The contents of OTC CBD preparations are of variable quality. A US study of 84 CBD products sold online in 2016 found that only 26/84 (31%) products accurately reported the amount and concentration of CBD that they contained.^8^ Vaporiser liquids were particularly inaccurate: only 3/24 (13%) products were correctly labelled. Moreover, that study found that 18/84 (21%) samples contained Δ^9^-THC, with a mean concentration of 0.45 mg/ml (standard deviation: 1.18; max: 6.4 mg/ml). More recent studies have shown that this continues to be a major problem. A 2020 study from Mississippi found that only 3/25 (12%) products were within 20% of what the label claimed and 3/25 (12%) had a Δ^9^-THC content exceeding legal limits.^91^ A UK study found that only 11/29 (38%) products had within 10% of the amount of CBD advertised and one product did not contain any CBD at all.^92^ Reports on CBD products from Switzerland,^2^ The Netherlands^93^ and other European countries^94^ have found similar results. There have even been reports of CBD-rich extracts containing enough Δ^9^-THC (3–4%) to intoxicate young children,^95^ as well as concerns that some products contain enough Δ^9^-THC for users to fail illicit drug tests.^96^

As well as cannabinoids, other contaminants have been found in OTC CBD products, including 5F-ADB and AB-FUBINACA, synthetic cannabinoid receptor agonists,^97,98^ dextromethorphan, an anti-cough medication and dissociative hallucinogen^97^ and pesticides.^99^ A recent case report described the admission of an otherwise healthy man to an intensive care unit after eating two packets of CBD gummies, although the responsible compound was never identified.^100^ Additional safety concerns include lung injury from CBD vaporizers,^101^ the use of CBD as an unproved alternative for established treatments for serious medical conditions,^102^ and the long-term effects of CBD in children and adolescents, given the role of the endocannabinoid system in neurodevelopment.^103^

## Legal aspects

The legal status of cannabidiol is complex. Many countries now allow the prescription of licensed medicines which contain CBD, such as Epidiolex, and others also permit the prescription of a broader range of cannabis-based medicinal products without specific licensed indications,^1^ for example, Bedrolite, which contains less than 1.0% Δ^9^-THC and 9% CBD 9%, and is available through the Dutch Office for Medicinal Cannabis.^104^ In Uruguay, Georgia, Canada and several US states, recreational cannabis use is legal, and in many other countries it has been decriminalised. In jurisdictions where it is legalised, users are often able to purchase strains of cannabis with high CBD and low Δ^9^-THC content from cannabis dispensaries.

OTC preparations of cannabidiol, the ‘cannabis oils’ bought in a health shop, are subject to a variety of different laws and regulations across the world. In the European Union (EU) and the UK, CBD oil has been classified as a ‘novel food’ since January 2019.^105^ Novel foods are defined as foods which have not been widely consumed by EU citizens before 15 May 1997. They are subject to regulation and cannot advertise medicinal benefit. The EU also requires that Δ^9^-THC levels in these products must be below 0.2%.^106^ Sweden has additional legislation banning the sale of any product containing Δ^9^-THC, while in Slovakia all products containing CBD are illegal. Previously, CBD was considered a medicinal product in many EU jurisdictions. For example, in 2016, the UK Medicines and Healthcare Products Regulatory Agency (MHRA) informed companies that they would need to ‘operate within the law, by withdrawing their existing products from the market, or working with MHRA to satisfy the legal requirements of the Human Medicines Regulations 2012’.^107^

CBD products are not available as OTC supplements in Australia or New Zealand as they require a prescription.^108,109^ In Australia, hemp products are legal, but must have minimal concentrations of both CBD (<75 mg/kg) and Δ^9^-THC (<50 mg/kg).^109^ In Canada, both recreational and medical cannabis have been legalised since 2018. Products containing CBD are subject to specific regulations on their production, Δ^9^-THC content, labelling and packaging.^110^ In the US, the 2018 Farm Bill removed hemp (defined as having a Δ^9^-THC concentration ⩽0.3%) from the Controlled Substances Act. However, CBD extracted from non-hemp cannabis strains remains a Schedule I Controlled Substance.^111^ The US Food and Drug Administration has made it clear that CBD products cannot be sold as food or dietary supplements and has sent warning letters to manufacturers of CBD products, informing them that their medicinal claims were illegal.^112^ The complex interaction between US state and federal law has been described elsewhere.^111^

## Conclusion

Although there is enormous consumer interest in CBD, there is little evidence that OTC preparations have significant pharmacological activity or provide health benefits. However, there have been relatively few studies that have explicitly sought to evaluate CBD at very low doses, or with the other constituents that are typically found in OTC preparations. Although the licensing of food supplements does not require demonstrations of their safety and efficacy, controlled trials of OTC preparations are needed to address this issue. There is also a need for more accurate labelling and advertising of OTC CBD products.

## Key points

- It is unknown if over-the-counter products can produce plasma concentrations of cannabidiol high enough to have significant pharmacological effects.
- There is little evidence that over-the-counter cannabidiol products have therapeutic benefits.
- Labelling of the constituents of over-the-counter cannabidiol products is often inaccurate.
- Some over-the-counter cannabidiol products contain Δ^9^-tetrahydrocannabinol, which has the potential to cause adverse effects and positive urinary drug results for cannabis.
- Controlled trials of over-the-counter products are needed to resolve these issues.
